# Honey Differentiation Using Infrared and Raman Spectroscopy Analysis and the Employment of Machine-Learning-Based Authentication Models

**DOI:** 10.3390/foods14061032

**Published:** 2025-03-18

**Authors:** Maria David, Camelia Berghian-Grosan, Dana Alina Magdas

**Affiliations:** 1 National Institute for Research and Development of Isotopic and Molecular Technologies, 67-103 Donat Street, 400293 Cluj-Napoca, Romania; maria.david@itim-cj.ro (M.D.); camelia.grosan@itim-cj.ro (C.B.-G.); 2 Faculty of Physics, Babeș-Bolyai University, Kogălniceanu 1, 400084 Cluj-Napoca, Romania

**Keywords:** honey composition, acacia honey authentication, geographical differentiation, IR spectroscopy, Raman spectroscopy, ML classification

## Abstract

Due to rising concerns regarding the adulteration and mislabeling of honey, new directives at the European level encourage researchers to develop reliable honey authentication models based on rapid and cost-effective analytical techniques, such as vibrational spectroscopies. The present study discusses the identification of the main vibrational bands of the FT-Raman and ATR-IR spectra of the most consumed honey varieties in Transylvania: acacia, honeydew, and rapeseed, exposing the ways the spectral fingerprint differs based on the honey’s varietal-dependent composition. Additionally, a pilot study on honey authentication describes a new methodology of processing the combined vibrational data with the most efficient machine learning algorithms. By employing the proposed methodology, the developed model was capable of distinguishing honey produced in a narrow geographical region (Transylvania) with an accuracy of 85.2% and 93.8% on training and testing datasets when the *Trilayered Neural Network* algorithm was applied to the combined IR and Raman data. Moreover, acacia honey was differentiated against fifteen other sources with a 87% accuracy on training and testing datasets. The proposed methodology proved efficiency and can be further employed for label control and food safety enhancement.

## 1. Introduction

Being one of the oldest natural sweeteners, whose appreciated taste and nutritional value have been acknowledged since antiquity, honey is a desired commodity that finds applications in modern medicine [1]. Due to its particular properties, honey has found applications not only as a sweetener but also in wound treatment or in diminishing symptoms of various diseases such as infections or ulcers [2,3]. Honey is still undergoing further testing to ascertain its potential as an adjuvant in treating different diseases [3,4]. The particular nutritional and medical properties of honey are associated with components specific to each botanical type and depend on the harvesting period or geographical origin [5,6]. The increased commercial interest in this superfood has led to numerous adulteration issues in the attempt of producers to gain illicit profit, despite that these practices are threatening consumers. As a result, many researchers are focusing on discouraging the unfair practices of adulteration [7], which can occur through the addition of syrups (corn, starch, etc.) [8] or by partial/total substitution of some special (i.e., sea buckhorn) or highly consumed (i.e., acacia and linden) types of honey with lower priced varieties for an illegal economic profit [9,10], through the development of honey authentication models. Other undesired fraudulent practices are related to the false declaration of honey’s botanical source and geographical origin. This not only poses unfair competition within the industry but also affects the chemical composition of honey, altering its flavor and potentially diminishing the induced health benefits [9]. In this regard, the European Parliament reached a provisional agreement to improve consumer transparency and information requirements for honey by stipulating, on the label, the countries of origin [11,12].

To ensure market standards, many effective authentication approaches in terms of botanical and geographical origin recognition have been conducted [13] based on melissopalynology [14] mass spectrometry [15,16,17,18] or isotopic [19,20,21,22] and elemental profiling [23,24,25]. As these methods require specialized personnel for sample preparation as well as for the data acquisition phase, other analytical approaches based on vibrational spectroscopies were employed to develop reliable honey authentication models as they are easy to use, have a fast response time, and are environmentally safe. In this regard, IR or Raman spectroscopies have been previously reported as powerful analytical techniques for ensuring food quality [26], founding applications in honey authentication and adulteration detection [13,27,28]. For the obtainment of reliable honey authentication models, in the previously reported studies, the vibrational spectra were analyzed using various chemometric methods such as Principal Component Analysis (PCA) [29,30,31], Soft Independent Modeling Class Analogy (SIMCA) [32], Partial Least Squares–Discriminant Analysis (PLS-DA) [33,34,35,36], and Linear Discriminant Analysis (LDA) [37], while other approaches are based on artificial intelligence algorithms [38,39].

The two spectroscopic tools, namely IR and Raman spectroscopy, are complementary techniques because depending on the molecule symmetry, vibrational transitions that are IR-active may be forbidden in Raman and vice versa. In this regard, IR spectroscopy measures the interaction of infrared radiation with matter by absorption, a vibration being considered IR-active if it induces a change in the dipole moment of the molecule, making it particularly sensitive to polar bonds. By contrast, Raman spectroscopy examines the vibrational modes of the molecules relying upon inelastic scattering, detecting vibrations that result in a change in the polarizability of the molecule, making the method more effective at highlighting non-polar bonds. Therefore, their combined data can yield a more comprehensive molecular fingerprint when analyzing complex matrices such as honey, which comprises over 200 major and minor compounds (i.e., carbohydrates, proteins, organic acids, polyphenols, vitamins, and minerals).

Although IR and Raman vibrational spectroscopies are complementary and can provide extensive information about the chemical composition of any food matrix [40,41], their fusion applied for developing robust honey authentication models has been scarcely discussed in the literature. In this regard, few studies discuss the fusion of these vibrational spectroscopies data with the information obtained from other analytical techniques, processed using supervised statistical methods to differentiate honey varieties [42,43] or to predict honey’s antioxidant activity [44]. Only two analytical methods, namely mid-infrared and Raman spectroscopy, were applied in studies addressing the adulteration issue [45]. More recently, a data fusion strategy based on these spectroscopic methods and Probabilistic Neural Networks or Support Vector Machines indicated that data fusion can improve the geographical discrimination of honey collected from five regions of China [46].

This article employed the green vibrational methods (IR and Raman spectroscopies) for the analysis of honey samples having distinct botanical and geographical sources. In this regard, the vibrational profiles of the most common honey varieties produced in Transylvania, a specific region of Romania, represented by acacia, honeydew, and rapeseed, were described. The identification of the characteristic bands present in the vibrational spectra was performed considering the chemical composition of honey. This study outlines the distinctions in the shape and intensity of vibrational bands by visual pattern recognition, attributing these variations to the molecular varietal-dependent composition of honey. Among these three honey varieties, acacia honey is one of the most appreciated types in Transylvania and other European Union (EU) countries, because of its unique organoleptic properties, distinguished flower flavor, and longer crystallization time, being considered a premium honey. Moreover, given that Romania is the third largest honey exporter from the EU—28 countries [47]—with Transylvania being recognized for its diverse floral resources, traditional beekeeping practices, and reputation for producing premium honey, it is an excellent case study for exploring geographical differentiation. In this regard, it is imperative to protect honey production against fraudulent practices such as the false labeling of the botanical and geographical source by implementing honey quality control models based on rapid analytical techniques.

In the frame of the present work, a step forward was taken in exploring the potential and effectiveness of combining IR and Raman vibrational data, and a new methodology for developing novel honey authentication models is proposed. In this regard, the current pilot study aims to differentiate acacia honey from other varieties and to distinguish honey produced in a restricted geographical area of Romania, Transylvania, from that produced in other geographical regions of the EU (Italy, Greece, Portugal, and France). To achieve this, the combined data obtained through the concatenation of IR and Raman spectra of the investigated honey were used due to the analytical methods’ complementarity that could enhance the honey classification models’ accuracies. The fused data were processed with several ML tools, some of which (i.e., the *Trilayered Neural Network* algorithm) were employed and reported as a premiere in food control studies. The authentication model’s accuracies demonstrated efficacy in classifying honey samples from specific botanical sources and collected within a limited geographical area. The workflow of the present pilot study can be presented to regulatory agencies that can further ensure honey label validation.

## 2. Materials and Methods

### 2.1. Sample Acquisition

A total number of 77 authentic honey samples obtained exclusively from recognized and trustworthy producers were used in the present study, and their IR and Raman spectra were recorded. From this, for visualizing the spectral differences between the three most consumed honey varieties in Transylvania, the IR and Raman spectra of 15 acacia, 10 honeydew, and 10 rapeseed honey samples were chosen and averaged.

For the development of botanical variety ML authentication models, 16 acacia honey samples were compared versus 61 other honey samples having the following botanical distribution: linden (12), rapeseed (10), honeydew (13), polyflower (7), chestnut (5), sunflower (2), raspberry (2), coriander (1), sea buckthorn (1), heather (2), hawthorn (1), orange (2), lavender (1), thistle (1), and thyme (1). Regarding the employment of the geographical origin differentiation model, 60 authentic honey samples from Transylvania and 17 honey samples collected from other EU countries, such as Greece (7), France (7), Italy (2), and Portugal (1), were analyzed. The EU foreign honey samples were gathered from reputed producers in Greece, France, Italy, and Portugal. This selection was made to cover two predominant climatic regions: the Mediterranean (Greece, Italy, and Portugal) and the temperate (France and Romania) zones, ensuring a diverse geographical representation for honey authentication.

### 2.2. Vibrational Spectra Collection

Before spectral acquisition, to avoid crystallization, the samples were heated at 35 °C and homogenized by stirring [36,38]. For each honey sample, IR and Raman spectra were recorded in triplicate and averaged to obtain the final spectrum. The Raman spectra of the 77 honey samples were determined by a Bruker, Equinox 55 FT-Raman spectrometer equipped with a Nd:YAG laser emitting at 1064 nm and a Ge detector cooled with liquid nitrogen. All spectra were recorded with a resolution of 4 cm^−1^ in the spectral range −1000 to 3600 cm^−1^ by the co-addition of 100 scans [36].

The IR spectra of all of the honey samples were recorded on a Jasco 4100 FTIR spectrometer using an attenuated total reflectance (ATR) tool. For each sample, a drop of honey was placed in order to cover the ZnSe crystal of the ATR, and the spectrum was recorded in the spectral range 600–4000 cm^−1^ with a resolution of 4 cm^−1^ by averaging 16 scans [38]. The air spectrum was taken as the background between sample spectra acquisition, and acetone was used to clean the ATR crystal.

**Figure 1:**
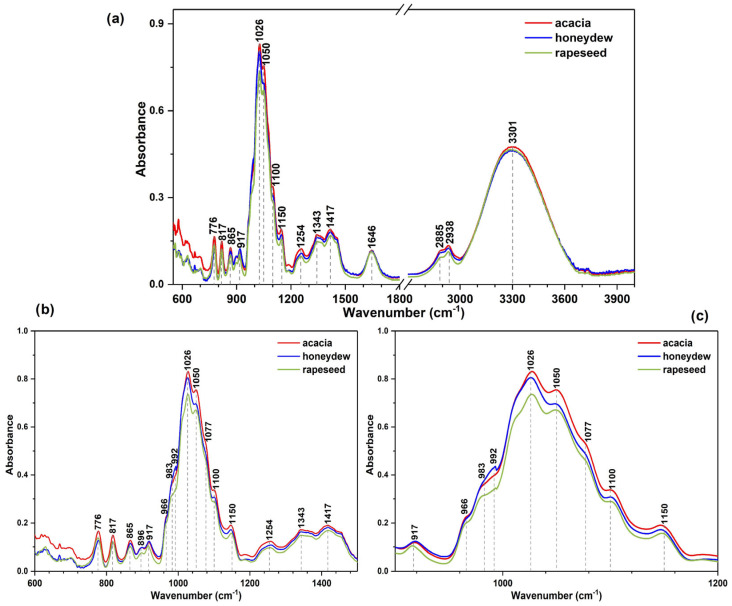
Average ATR-IR spectra of the *acacia*, *honeydew,* and *rapeseed* honey samples in the (**a**) spectral region 600–1800 and 2700–4000 cm^−1^ (the spectral break excluded the region presenting no significant signals for honey characterization, being affected by air), (**b**) 600–1500 cm^−1^, and (**c**) 900–1200 cm^−1^ (to better illustrate the visual differences among the investigated honey varieties, a normalization to [0,1] followed by a baseline correction was performed).

**Figure 2:**
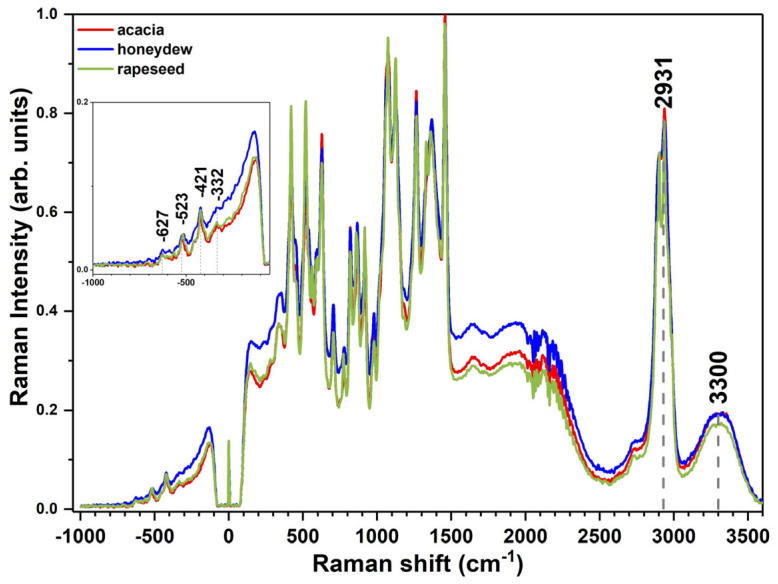
Comparison of the average Raman spectra of the investigated honey varieties in the entire measured spectral range (to better illustrate the visual differences among the investigated honey varieties, a normalization to [0,1] was performed).

**Figure 3:**
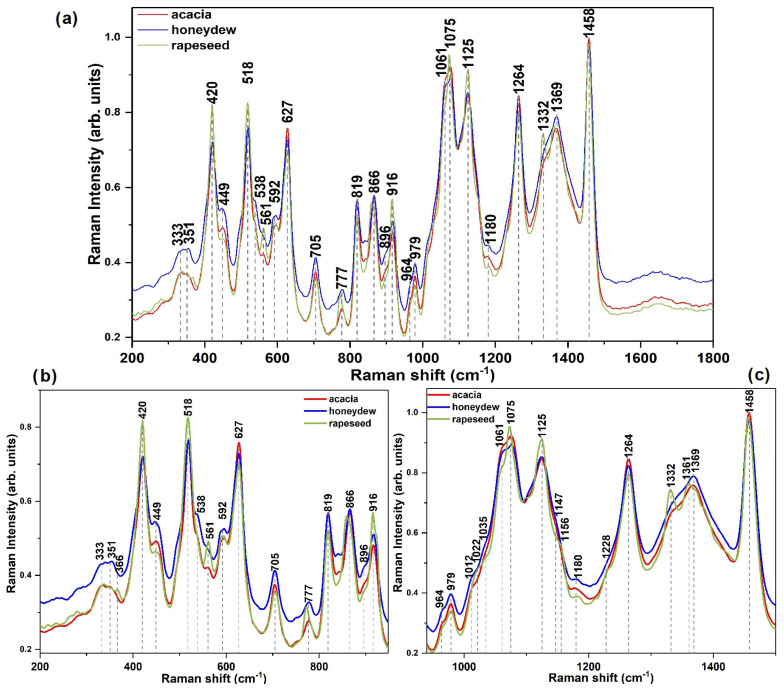
Average FT-Raman spectra of the honey samples having acacia, honeydew, and rapeseed as botanical sources in the (**a**) whole fingerprint region, (**b**) 300–950 cm^−1^, and (**c**) 900–1600 cm^−1^ (to better illustrate the visual differences among the investigated honey varieties, a normalization to [0,1] was performed).

The OriginPro 2017 software was used to plot the IR and Raman spectra for each honey variety (*acacia*, *honeydew*, and *rapeseed*). For a better visualization and presentation of the experimental data (Figure 1, Figure 2 and Figure 3), a normalization to [0,1] was applied [38,48]. In the case of the IR spectra, an additional baseline correction was performed, only for visualization reasons.

For the ML model development based on IR and Raman data, the experimental spectra were used without any preprocessing treatment.

### 2.3. Machine Learning Investigation

The classification models were obtained using the Classification Learner app from Matlab R2024a (MathWorks, Natick, MA, USA). In total, 34 types of classifiers (Trees, Discriminants, Naive Bayes, Support Vector Machines—SVM, K-Nearest Neighbors (KNN), Ensembles, Neural Networks, and Kernels) were employed and compared regarding their performance on both training and testing datasets. The datasets were created using the combined vibrational data (IR and Raman spectra) of the 77 honey samples and subsequently split into groups in accordance with the two types of discrimination studies. The test set was chosen in a stratified manner to maintain the distribution of the investigated groups (i.e., acacia/other varieties; Transylvania/other EU countries). Thus, for the differentiation of the acacia honey from fifteen other varieties (i.e., rapeseed, honeydew, linden, polyflower, sunflower, chestnut, raspberry, orange, sea buckthorn, heather, hawthorn, coriander, thistle, and thyme), the sample set was divided into two subsets, 70% of the samples being used for training and validation and 30% of the samples (comprising 5 acacia, 5 polyflower, 3 linden, 3 chestnut, 2 honeydew, 1 orange, 1 raspberry, 1 heather, 1 sunflower, and 1 rapeseed honey) solely for testing. The recognition of honey samples of Transylvanian origin from the samples coming from Greece, France, Italy, or Portugal made use of a training set containing about 80% of the total number of samples (48 samples from Transylvania and 13 samples from other EU countries) and a testing set representing 20% of the total number of samples (12 samples from Transylvania, two samples from France, one sample from Greece and one sample from Italy). Moreover, for these sets’ preparation, the fusion of data from the regions 600–1800 cm^−1^ of the IR spectra and 200–1799 cm^−1^ of the Raman spectra was considered. The 6-fold cross-validation evaluation procedure was selected for both discrimination studies, and the best prediction models were chosen by considering both the training and testing accuracy values.

The performance of each classifier, for the two differentiation models (botanical and geographical), are presented in Tables S1 and S2. In the case of the botanical origin assessment model that is capable of discriminating acacia honey from other types, the Ensemble subspace discriminant algorithm proved to be the most performant, as will be discussed in Section 3.3, with a number of 30 learners and the subspace dimension of 1038. For the geographical source differentiation, the Trilayered Neural Network model proved to be the most efficient in correctly classifying Transylvania-produced honey from that which originated from other EU countries, achieving the highest accuracies on both training and testing datasets. The model hyperparameters are as follows: number of fully connected layers—3, first layer size—10, second layer size—10, third layer size—10, activation—ReLu, iteration limit—1000, regularization strength (Lambda)—0, and standardized data—yes.

## 3. Results and Discussion

The current study aims to identify the unique characteristics of the IR and Raman spectra of the three predominant honey varieties produced and consumed in Transylvania, a region of Romania, namely *acacia*, *honeydew,* and *rapeseed*, through a visual assessment of the variations observed in the samples’ average spectra. In this regard, the spectra were compared and the differences in the vibrational features were explained based on the particular chemical composition of the investigated honey types. Following this evaluation, the regions of the vibrational spectra exhibiting the most significant variances associated with the specific varietal-dependent compounds were chosen as input data for a pilot study aiming at the development of honey recognition models that use the combined IR and Raman data processed with ML algorithms.

As illustrated in Figure 1, Figure 2 and Figure 3, the main vibrational bands observed in the IR and Raman spectra of the investigated samples correspond to the major compounds present in honey, namely water and sugars. This is because honey is defined as a syrupy solution that consists mainly of water and carbohydrates, with glucose, fructose, and sucrose present in the highest concentrations. The concentration distributions of these major compounds were previously reported as an important criterion for the botanical and geographical classification [49,50]. Still, the emergence of new bands or changes in the position (shifts) of the vibrational bands of the corresponding pure compound may be detected due to the presence of other honey components at significantly lower concentrations, such as proteins, free amino acids, organic acids, flavonoids, vitamins, minerals, and several volatile compounds, which contribute to the organoleptic properties of honey [36,51].

### 3.1. ATR-IR Spectra Analysis

In order to visually observe the differences that may occur in the ATR-IR spectra of the most common Transylvanian honey samples having *acacia*, *honeydew*, and *rapeseed* as botanical origins, the spectral data were collected in the 600–4000 cm^−1^ domain. For better discrimination among distinct spectral fingerprints, the spectra were normalized and a background subtraction was employed. The average ATR-IR spectra of the analyzed honey samples can be observed in Figure 1.

The comprehensive band assignment of the ATR-IR honey spectra is presented in Table 1, incorporating a data collection of the existing literature. The IR fingerprint region, situated between 600 and 1800 cm^−1^ [30,38], is characterized by wide bands mostly associated with the vibrations of sugars (mostly glucose, fructose, and sucrose), as well as water. Identification of the minor components, such as proteins, amino acids, vitamins, and organic acids, in the honey spectra can be achieved by the detection of shoulder-type signals or by observing slight positional shifts of the bands [52]. The region between 600 and 700 cm^−1^, known as the “carbon skeletal vibration” area, is, in this case, slightly affected by noise (Figure 1a).

The anomeric region of carbohydrates can be observed in the 700–950 cm^−1^ domain, whereas the 950 to 1200 cm^−1^ region presents the C–O, C–C stretching vibrations of carbohydrates alongside some signals from proteins around 1077 cm^−1^ (Table 1). Several differences among the average spectrum of each honey variety are indicated in Figure 1c, including (*i*) band shifts (e.g., from 983 to 992 cm^−1^); (*ii*) variations in peak intensities (e.g., the signals around 1026, 1050, 1100, and 1146 cm^−1^), as well as (*iii*) peak shape modifications (notably observed for the band around 992 cm^−1^). The disparities can be explained by the variations in sugar content among the samples, as glucose presents an absorption peak at 992 cm^−1^, while the band at about 983 and 1100 cm^−1^ correspond to sucrose stretching of the C–O bond and the C–O–C linkage [52]. The same order of the absorbance intensity is consistent for the bands between 1200 and 1350 cm^−1^, which were also reported to be dependent on the sucrose content [53]. The observable variations in the IR absorption intensities among samples are correlated with sucrose concentrations, which differ according to the honey type [50], and can be a possible explanation for the spectral differences observed in the case of the honey samples analyzed in the present study. The absorption peak at 1150 cm^−1^, characterized by varying maxima, can be attributed to maltose’s vibrational modes, a varietal-dependent disaccharide [54]. Moreover, the region between 1040 and 1106 cm^−1^, corresponding to the C–H third overtone, presents notable intensity variations among the average spectra of the samples and has been previously reported as being proportional to the concentration of glucose present in spiked honey samples [55].

Other differences that can be noted in the IR spectra of the analyzed samples are present in the region between 1200 and 1500 cm^−1^, which is suggestive of the symmetric and asymmetric bending of the C–H bands in carbohydrates, as well as the presence of proteins due to the amide III vibration mode (involving the wagging of N–H, C–O and the stretching of C–C, C–N) [56,57,58]. The subsequent broad IR bands exhibited at 1600–1700 cm^−1^ can be correlated with the stretching vibration of the O–H bond in water, C=O in carbohydrates, and N–H bending of proteins [53,56,57]. The C–H stretching vibrations of carbohydrates, O–H from carboxylic acids, NH_3_ of amino acids, and the stretching vibration of the O–H bonds can be found in the region 2800–3600 cm^−1^ [30,52,56,57]. This implies that the different quantities of carbohydrates, along with the presence of proteins and amino acids, influence the unique chemical composition of the investigated honey varieties, as evidenced in the IR spectra.

Overall, the IR signals present between 600 and 1800 cm^−1^ are the most sensible spectral areas, being highly affected by variations in the concentrations of carbohydrates, amino acids, organic acids, and vitamins, which are particular to each investigated honey variety (i.e., acacia, honeydew, and rapeseed). Thus, the characteristic vibrations of specific constituents such as glucose, fructose, sucrose, organic acids (i.e., gluconic, citric, lactic, and fumaric), riboflavin, ascorbic acid, L-cystine, and many others appear as signals in these regions, with their ratios being directly influenced by the botanic source of honey [54].

**Table 1:** Band assignments of the corresponding vibration modes present in the ATR-FTIR spectra of the investigated honey samples.

ATR-IR Band Position (Intensity) in cm^−1^	Assignment of The Vibrational Mode	Refs.
	Fingerprint Region	
600–700 (vw)	“crystalline region” containing the exocyclic deformations of (CCO) and endocyclic deformations of (CCO, CCC)	[30,52,59]
		
776 (w)	Anomeric region of carbohydrates; C–H bending vibrations; ring vibrations; and COH, CCH, OCH bond bending	[52,60]
817 (w)		
856 (w)		
917 (w)		
		
966 (m)	C–O (from C–OH) and C–C stretching in carbohydrates	[30,52]
983 (m)		
992 (m)		
		
1026 (s)	C–O stretching in carbohydrates	[52,54,61]
1050 (s)		
		
1077 (m)	C–N stretching in proteins	[30,52,56,57]
1100 (m)	The stretching of the C–O bond of the C–O–C linkage	[30,52]
		
1150 (m)	C-CO-C bending and stretching in carbonyl group, presence of maltose	[54,61]
		
1254 (w)	Amide III vibration mode	[56,57,59]
1343 (w)	O–C–H, C–C–H, and C–O–H bending modes	[30,52]
		
1417 (w)	Carbohydrate C–H stretching band, C–H bending of alkenes, and a CH=C Vibration mode of organic acids such as fumaric acids	[30,52]
		
	Stretching vibrations area	
Broad band (m) with a maximum peak at 1646	O–H stretching/bending of water, C=O stretching mainly from carbohydrates, and N–H bending of amide I from proteins	[52,61]
		
Broad band (m) with a maximum peak at 2885 and 2938	C–OH mode in carbohydrates, O–H stretching from carboxylic acids, and NH_3_ stretching from amino acids	[30,52,61]
		
Broad band (s) with a maximum peak at 3301	O–H stretching	[30,61]

### 3.2. FT-Raman Spectra Analysis

The Raman spectra of the acacia, honeydew, and rapeseed honey were recorded from −1000 to 3600 cm^−1^ in both the Stokes and anti-Stokes regions. The signals from the anti-Stokes region emerge from molecules in vibrational excited states during Raman excitation. As the majority of the molecules in honey are in the ground state, their vibrations will appear in the Stokes region at frequencies between 200 and 3600 cm^−1^. In Figure 2, the whole Raman spectra of the samples can be observed. Specific regions can be delimited in the Stokes region: the fingerprint domain between 200 and 1600 cm^−1^ characterized by an increased number of signals; a low-intensity signal region between 1800 and 2500 cm^−1^ affected by the fluorescence of the samples (especially present in the case of the honeydew samples, which are darker in color and known for their fluorescence effect); and prominent broad bands near 2900–3100 cm^−1^ and 3300 cm^−1^. The assignment of Raman spectral bands is provided in Table 2.

The anti-Stokes region (Figure 2) exhibits significant signals at −627, −523, −421, and −332 cm^−1^ which, according to the literature, can be attributed to the specific vibrations of saccharides (glucose and fructose), organic acids, and proteins [62,63]. This domain was successfully employed for the authentication of other food matrixes, such as wine [64].

The fingerprint region between 200 and 1800 cm^−1^ (Figure 3) comprises intense signals resulting from the scattering vibrations of the major components: glucose, fructose, and sucrose. In honey samples, unlike the case of the pure components, the bands associated with these compounds are shifted [63] or may exhibit shoulder-type signals belonging to the other minor components of honey. Thus, the intensity of some Raman peaks, specifically at 351, 420, 518, 561, 592, 627, 705, 777, 819, 866, 916, and 979 cm^−1^, as well as those in the 1000–1200 cm^−1^ region and 1332 cm^−1^ are dependent upon the quantity of the components’ functional groups. The signal intensities at 351, 420, and 1075 cm^−1^ were previously reported to be influenced by compounds including proteins, amino acids, and organic acids [65]. The other observed variances are mostly due to the ratios of glucose, fructose, and sucrose present in honey samples having different floral origins.

Overall, the spectral range between 1000 and 1600 cm^−1^ is crucial for identifying the sugar presence as it exhibits the stretching vibrations of C–OH, C–C, and the bending of C–O–H of the specified carbohydrates. The bands characteristic to glucose are located at 705, 777, 916, 1022, and 1125 cm^−1^, whereas the bands specific to fructose are situated at 627, 819, 866 cm^−1^, the shoulder of the 926 cm^−1^ band, as well as at 979, 1061, 1264, 1332, and 1458 cm^−1^ [66]. The band corresponding to the most abundant disaccharide in honey, namely sucrose, appears at 1361 cm^−1^. Moreover, it can be observed that the bands associated with glucose present an increased intensity in the case of rapeseed honey as compared to the acacia one, aligning with previously reported studies that indicate higher amounts of glucose in the Romanian rapeseed honey [67].

Other variations among samples derived from acacia, honeydew, and rapeseed are evident as shoulders within the bands from 627, 1126, and 1458 cm^−1^, which can be correlated to the C–N group vibrations of amino acids and proteins, whereas the band at 1264 cm^−1^ can be associated with the vibrational modes of peptides (amide III) [65]. The carbonyl group stretching vibrations exhibit an absorption band between 1600 and 1800 cm^−1^ [57], indicating the presence of some volatile compounds including aldehydes and ketones that are associated with the botanical source of honey [68].

The broad peaks at 2931 and 3300 cm^−1^ encompass the C-H stretching vibration and asymmetric stretching of CH_2_ in addition to the O–H stretching vibration from water [30,61,67].

**Table 2:** Raman band position and compound attribution based on the chemical composition of honey samples.

FT-Raman Band Position (Intensity) in cm^−1^	Assignment of the Vibrational Mode	Refs.
	Region 1—Anti-Stokes Region	
−523 (w)	Glucose	[62,63]
−627 (w)	Fructose	
		
	Region 2—Fingerprint Area	
333 (w)	Sucrose’s glycosidic linkage C–O–C bending mode	[63,65]
351 (w)	Presence of glucose	[62,65]
420 (m)	Presence of glucose	[62,65]
449 (w)	Skeletal vibration	[65]
518 (m)	Presence of sucrose and fructose	[66]
538 (m)	Bending of C–C–O	[65]
592 (m)	Skeletal vibration	[65]
627 (m)	Ring deformation vibration, fructose bending of C–C–O (exocyclic)	[66]
705 (w)	Stretching vibration of CO, CCO, OCO, presence of fructose	[62,69]
777(vw)	Deformation of C–H in fructose	[62]
819 (m)	Presence of fructose	[62]
866 (m)	Presence of fructose	[62]
896	Bending of CH	[62]
916 (w)	Bending mode of CH and COH in glucose	[65]
979 (w)	Presence of anomeric fructose	[62,65,69]
1022 (shoulder)	Stretching vibrations of C–C and C–O from glucose	[65]
		
1061 (s)	Stretching vibration of C–OH, C–C, bending of C–O–H	[66]
1075 (s)		
		
1125 (s)	Bending C-OH, presence of glucose	[65]
1264 (s)	Bending vibration of CH, CH_2_, COH in crystallized fructose	[66]
1332	Presence of fructose	[65]
		
1361/1369 (m)	Bending in-plane asymmetric vibration of CH_2_, presence of disaccharides (sucrose)	[66]
1458 (s)	Bending vibration of CH, symmetric in-plane CH_2_, C–O–H from fructose	[66]

As a general observation, the Raman spectra of the investigated authentic honey varieties harvested in the Transylvania region of Romania, having acacia, honeydew, and rapeseed as floral sources, revealed bands both in anti-Stokes and Stokes regions that correspond to various honey components. In contrast to ATR-IR spectra, in the case of FT-Raman spectra, more noticeable differences may be noted in relation to the botanical origin of honey, such as variations in Raman peak intensities, the formation of shoulder-type signals, or shifts in band position. Considering these results, it can be concluded that the observed variances among the discussed honey types are derived from the distinct concentrations of sugars, as well as from the varying minor components including proteins, amino acids, and organic acids, whose concentrations are dependent upon the varietal source.

As IR spectroscopy presents absorption bands specific to the molecular vibrations that involve a change in the dipole moment, the Raman bands are obtained due to the inelastic scattering of induced radiation, which corresponds to a shift in energy induced by the change in polarizability of the molecules [56,63]. Their obtained spectral data are complementary and can be fused to have an assembly of the variables offered by the two spectroscopic analyses. Moreover, IR spectroscopy is suitable for honey analysis as the fluorescence effect in the spectra is eliminated as compared to the Raman spectra that can be influenced by this effect, while in water-based matrixes (such as honey), Raman spectroscopy can be more appropriate due to its weaker water bending mode, which does not interfere with the fingerprint region as significantly as IR spectroscopy [56,63].

The observed variances in the IR and Raman spectra of honey samples from different botanical sources facilitated the identification of the spectral region containing significant data about the unique compounds and their quantities that differentiate the botanical origins of honey. In this light, the IR region from 600 to 1800 cm^−1^ and the Raman domain from 200 to 1800 cm^−1^ were determined to be the most significant. The identified spectrum regions and the observation of the complementary nature of these spectroscopies have been used to construct models for honey authentication that may effectively differentiate acacia honey from other varieties. The same methodology was employed to construct geographical differentiation models that may successfully distinguish the particular honey production region of Transylvania from other EU regions. The pilot study that will be further detailed evaluates, for the first time, the potential and efficacy of employing both IR and Raman vibrational data to create honey authentication models based on botanical and geographical origins, thereby offering a comprehensive method for honey characterization. The proposed methodology may be presented to regulatory bodies for honey label validation through a rapid, sustainable analytical approach, which combines vibrational data to enhance the model’s discriminative capability by integrating complementary spectral features and relies on machine learning algorithms for the precise differentiation of honey samples.

### 3.3. Use of Machine Learning Algorithms for Honey Samples Evaluation

For the development of authentication models, a new methodology was analyzed. In this regard, we evaluated the efficacy of the proposed workflow by implementing two classification models designed to (*i*) differentiate the highly appreciated acacia premium honey, which is particularly susceptible to adulteration, and (*ii*) to distinguish Transylvania honey, a region renowned for rich floral diversity, traditional beekeeping methods, and a reputation for producing high-quality honey. Thus, considering the vibrational characteristics previously discussed and following our prior findings which demonstrated that both ATR-FTIR and FT-Raman data can be individually used in combination with machine learning algorithms for the successful development of honey recognition models (i.e., botanical or geographical origin differentiation or honey mixtures evaluation) [25,32,48], two types of ML-based analyses have been performed on a set of 77 honey samples. The first investigation assesses the performance of identifying acacia honey from many other varieties of honey (i.e., rapeseed, honeydew, linden, polyflower, sunflower, chestnut, raspberry, orange, sea buckthorn, heather, hawthorn, coriander, thistle, thyme, and lavender), whereas the subsequent investigation aims at the differentiation of Transylvanian honey in relation to samples from Greece, France, Italy, or Portugal. Unlike former studies, the current one overviews if the correlation between spectral data from two vibrational techniques, namely IR and Raman, enhances the accuracy of authentication models and assesses if the proposed methodology could provide a foundation for ensuring label control. In the case of both classifications, firstly, the vibrational data comprising the most significant information as highlighted in Section 3.1 and Section 3.2, namely the region from 600 to 1800 cm⁻^1^ for IR spectra and 200 to 1800 cm⁻^1^ for Raman spectra, were combined. Then, the fused data were processed, and the performance of the 34 algorithms available in the Matlab R2024a app (such as Trees, Discriminants, Naive Bayes, SVM, KNN, Ensembles, Neural Networks, and Kernels) was evaluated and further compared, Tables S1 and S2.

**Figure 4:**
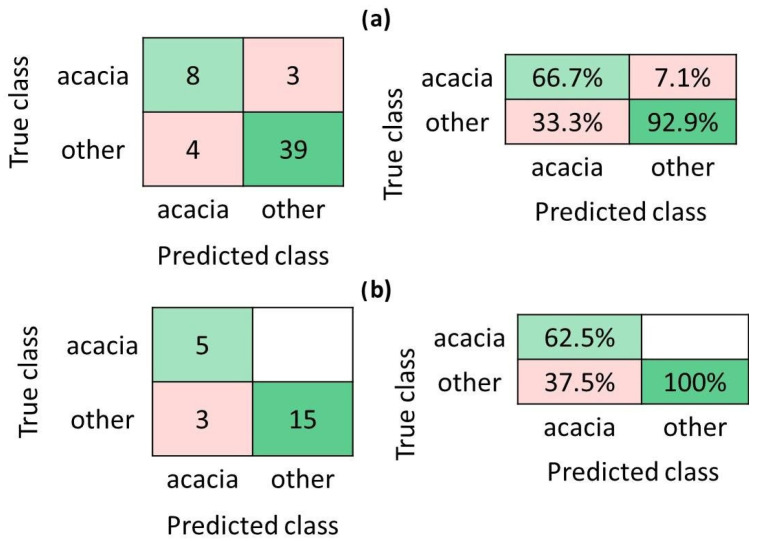
Acacia honey differentiation study (strategy: training 70%, testing 30% of samples). Confusion matrix, indicated as number of observations or precision scores and false discovery rates for (**a**) training and (**b**) testing data. *Ensemble subspace discriminant model* performance—accuracy 87%, both on training and testing datasets, when 6-fold cross-validation was considered.

For the first investigation, which identifies acacia honey, the binary GLM Logistic Regression model leads to the smallest accuracy value of 55.6% for training investigation while, on the testing set, the smallest accuracy value of 47.8% is obtained for the fine and medium tree models. Figure 4 indicates the performance of the most effective classification model, an *Ensemble subspace discriminant model*, in training (Figure 4a) and testing (Figure 4b). Analyzing these outcomes, it appears that few samples, from both classes, were wrongly associated; thus, three *acacia* samples were assigned to the *other* honey varieties while four samples of the class *other* were classified as *acacia* ones, Figure 4a, yielding an average accuracy value of 87% in six-fold cross-validation, a precision of 66.7% and 92.9%, and false discovery rates of 33.3% and 7.1% for *acacia* and *other* classes, respectively, Figure 4a.

The evaluation of the *Ensemble subspace discriminant model* on the testing dataset showed an accuracy value of 87%, with only 3 out of 23 honey samples of the class *other* being wrongly classified as *acacia* ones. The model was able to correctly recognize all the acacia honey samples contained in the testing set, although, overall, a false discovery rate of 37.5% can be noted for acacia samples because of the false positives identified here, Figure 4b.

It is worth mentioning that the *Ensemble* classifier (*bagged trees* or *subspace discriminant*) was also identified, in another study, as yielding the highest accuracy for a honey differentiation model in relation to the botanical origin when the Raman spectra (without any pretreatment) of several honey varieties, namely acacia, chestnut, colza, lavender, linden, sunflower [33], or acacia, colza, honeydew, linden, and raspberry, were employed [48]. The present pilot study obtained performant results when the honey varietal distribution was further extended compared to the previous studies and was capable of differentiating acacia honey from fifteen other honey varieties, with an accuracy of 87% on both the training and testing dataset.

**Figure 5:**
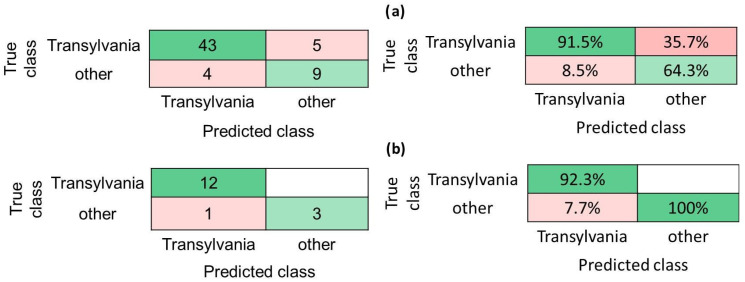
Discrimination of honey samples having a Transylvanian origin (strategy: training 80%, testing 20% of samples). Confusion matrix, indicated as number of observations or precision scores and false discovery rates for (**a**) training and (**b**) testing data. *Trilayered Neural Network model* performance—accuracy of 85.2% and 93.8% on training and testing datasets, respectively, when 6-fold cross-validation was considered.

For the second study concerning the honey samples differentiation in regard to their geographical origin, our study focused on discriminating samples from the Transylvania region of Romania from all other samples (which contain honey originating from Greece, France, Italy, or Portugal). In this regard, 80% of the samples were used for creating and validating the prediction models, while 20% were solely employed as test instances for an unbiased evaluation of the classifiers. This distribution was made because of the ratio between the numbers of samples in the two classes. As for the previous investigation, 34 classifiers, such as Trees, Discriminants, Naive Bayes, SVM, KNN, Ensembles, Neural Networks, and Kernels, were tested to build the final geographical recognition model. The results are presented in Table S2 and indicate a significant variation between the models in relation to the accuracy values; thus, the Logistic Regression shows the smallest accuracy values of 49.2% and 25%, obtained on the training set (binary GLM Logistic Regression) and testing dataset (Efficient Logistic Regression), respectively. It is worth mentioning that several models lead to the same accuracy of 85.2% during the training tests (i.e., Trees, Linear SVM, Ensemble subspace discriminant, Ensemble—subspace KNN, Wide Neural Network, and Trilayered Neural Network), Table S2. Among these algorithms, the *Trilayered Neural Network model* conducted the highest performance in cross-validation on the testing dataset, having an accuracy of 93.8%, Table S2 and Figure 5. Therefore, for the training study, the model’s precision was 91.5% for the Transylvania class and 64.3% for the other class. In total, 9 samples (5 from the *Transylvania* class and 4 from the *other* class) out of the 61 used in this analysis were wrongly assigned; in this way, the percentages for false discovery rates became 8.5% (the *Transylvania* class) and 35.7% (the *other* class). It is worth mentioning here that the high value of the false discovery rate attributed to the class *other* is related to the low number of samples of *other* origins involved in the study, Figure 5a.

However, the results obtained after performing the model evaluation on the testing dataset indicate a good model performance—an accuracy of 93.8%, with a precision of 92.3% and 100% for the Transylvania and other class, respectively. Thus, the *Trilayered Neural Network* model was able to correctly assign all the Transylvanian honey samples employed in the training set; only one sample from the *other* class was attributed to the *Transylvania* one, this conducting to a false discovery rate for the *Transylvania* class of 7.7%, Figure 5b.

In this context, it is noteworthy that in a previous study that tested the possibility of classifying honey samples concerning their geographical origin [32], the best result achieved an accuracy of 82.7%, with the cubic SVM on honey samples originating from two far enough countries, Romania and France. In that case, the Raman spectroscopic data were used and the model evaluation performance indicated only one wrong classification, namely a French honey sample was assigned to the Romania class. However, Neural Networks were not employed at that moment for constructing the model [32]. Another study reported a slightly higher accuracy of 95% on the testing dataset for differentiating honey samples collected from five regions of China when the data from IR and Raman spectroscopy were fused and the data were processed using an optimal SVM model [46]. In that study, a higher number of 142 honey samples were employed for the construction of the model [46], while for the present study, only 77 samples were used. Moreover, this research tackles a more complex challenge of international honey differentiation across several EU regions. The comparable accuracies, despite the challenges posed by a reduced dataset and the broader geographical variability of samples, could highlight the robustness of the present honey authentication model. Higher accuracies could be obtained by applying the same methodology presented in this study on an expanded dataset that will also consider honey from other EU countries.

## 4. Conclusions

For the present study, two spectroscopic methods, namely ATR-IR and FT-Raman, were applied for analyzing and determining the spectral characteristics of the investigated honey samples, emphasizing the vibrational signature of acacia and Transylvanian honey. The band assignment of the acquired ATR-IR and FT-Raman spectra was conducted with regard to acacia, honeydew, and rapeseed honey types, which are the most esteemed Romanian honey varieties. The results gained from identifying the main vibrational signals indicated that possible differences between these honey types may derive from variances in chemical composition, encompassing both sugar profiles and minor components. Additionally, this pilot study proposed differentiation models capable of distinguishing a specific honey variety (acacia) from various other honey types, achieving 87% in both training and testing by employing the association between the two vibrational spectroscopic data and the *Ensemble subspace discriminant model*. The *Trilayered Neural Network model* was conducted to accuracies of 85.2% and 93.8% in training and testing, respectively, when distinguishing honey produced in a narrow region of Romania (Transylvania) from that collected in other EU countries. In both classification studies, the datasets were constructed through the concatenation of the data from the regions 600–1800 cm^−1^ of the IR spectra and 200–1799 cm^−1^ of the Raman spectra, based on the subsequent observation that these spectral regions comprise the vibrational modes of the most important compounds of honey. The results proved that the proposed methodology of the pilot study can be used for authenticating the highly valued acacia honey variety and a narrow region of honey production (Transylvania), yielding high accuracies in training and testing. The same workflow can be further applied to an expanded dataset involving more honey samples for training and testing of the authentication models. The outcome could be a valuable resource to ensure accurate labeling by the regulatory agencies as it employs fast analytical tools and accessible algorithms.

## Data Availability

Data will be made available upon request.

## Supplementary Materials

The following supporting information can be downloaded at https://www.mdpi.com/article/10.3390/foods14061032/s1: Table S1: Machine learning results obtained, using various algorithms and 6-fold cross-validation, for discrimination of acacia samples from other honey samples (bold—the better accuracy values obtained on the training and testing datasets); Table S2: Machine learning results obtained, using various algorithms and 6-fold cross-validation, for the Transylvanian origin investigation (italic—the better accuracy values obtained on the training dataset, bold—the better accuracy values obtained on the training and testing datasets).
