# Supplementary material for: Honey Differentiation Using Infrared and Raman Spectroscopy Analysis and the Employment of Machine-Learning-Based Authentication Models
Source: Foods. 2025 Mar 18;14(6):1032. doi: 10.3390/foods14061032 (PMC11941707; doi:10.3390/foods14061032)
Supplement: Supplementary file 1 [file foods-14-01032-s001.zip › foods-3491231-supplementary.pdf]

Supplementary Material

Honey Differentiation Using Infrared and Raman Spectroscopy Analysis and the Employment of Machine-Learning-Based Authentication Models

Maria David [1,2], Camelia Berghian-Grosan [1] and Dana Alina Magdas [1,2,*]

[1] National Institute for Research and Development of Isotopic and Molecular Technologies, 67-103 Donat Street, 400293 Cluj-Napoca, Romania; maria.david@itim-cj.ro (M.D.); camelia.grosan@itim-cj.ro (C.B.-G.)
[2] Faculty of Physics, Babeș-Bolyai University, Kogălniceanu 1, 400084 Cluj-Napoca, Romania
* Correspondence: alina.magdas@itim-cj.ro

Table S1. Machine learning results obtained, using various algorithms and 6-fold cross-validation, for discrimination of acacia samples from other honey samples (*bold*—the better accuracy values obtained on the training and testing datasets).

Botanical (acacia) origin investigation			
Model number	Model name	Accuracy values (%)	
		Training	Testing
2.1	Fine tree	64.8	47.8
2.1	Fine tree	64.8	47.8
2.2	Medium tree	64.8	47.8
2.3	Coarse tree	64.8	65.2
2.4	Linear Discriminant	75.9	82.6
2.5	Quadratic Discriminant	Failed	
2.6	Binary GLM Logistic Regression	55.6	65.2
2.7	Efficient Logistic Regression	79.6	78.3
2.8	Efficient Linear SVM	70.4	78.3
2.9	Gaussian Naive Bayes	70.4	52.2
2.10	Kernel Naive Bayes	77.8	73.9
2.11	Linear SVM	77.8	78.3
2.12	Quadratic SVM	85.2	73.9
2.13	Cubic SVM	81.5	73.9
2.14	Fine Gaussian SVM	79.6	78.3
2.15	Medium Gaussian SVM	79.6	78.3
2.16	Coarse Gaussian SVM	79.6	78.3
2.17	Fine KNN	79.6	65.2
2.18	Medium KNN	74.1	73.9
2.19	Coarse KNN	79.6	78.3
2.20	Cosine KNN	79.6	73.9
2.21	Cubic KNN	77.8	73.9
2.22	Weighted KNN	75.9	82.6
2.23	Ensemble—boosted trees	79.6	78.3
2.24	Ensemble—bagged trees	75.9	65.2
2.25	**Ensemble subspace discriminant**	**87.0**	**87.0**
2.26	Ensemble—subspace KNN	72.2	65.2
2.27	Ensemble—RUSBoosted trees	81.5	52.2
2.28	Narrow Neural Network	79.6	73.9
2.29	Medium Neural Network	81.5	69.6
2.30	Wide Neural Network	83.3	65.2
2.31	Bilayered Neural Network	77.8	56.5
2.32	Trilayered Neural Network	74.1	78.3
2.33	SVM kernel	79.6	78.3
2.34	Logistic Regression kernel	79.6	78.3

Table S2. Machine learning results obtained, using various algorithms and 6-fold cross-validation, for the Transylvanian origin investigation (*italic*—the better accuracy values obtained on the training dataset, *bold*—the better accuracy values obtained on the training and testing datasets).

Botanical (acacia) origin investigation			
Model number	Model name	Accuracy values (%)	
		Training	Testing
2.1	*Fine tree*	*85.2*	75.0
2.1	*Medium tree*	*85.2*	75.0
2.2	*Coarse tree*	*85.2*	75.0
2.3	Linear Discriminant	70.5	75.0
2.4	Quadratic Discriminant	Failed	
2.5	Binary GLM Logistic Regression	49.2	56.2
2.6	Efficient Logistic Regression	78.7	25.0
2.7	Efficient Linear SVM	68.9	75.0
2.8	Gaussian Naive Bayes	67.2	56.2
2.9	Kernel Naive Bayes	73.8	68.8
2.10	*Linear SVM*	*85.2*	81.2
2.11	Quadratic SVM	83.6	81.2
2.12	Cubic SVM	83.6	68.8
2.13	Fine Gaussian SVM	78.7	75.0
2.14	Medium Gaussian SVM	78.7	75.0
2.15	Coarse Gaussian SVM	78.7	75.0
2.16	Fine KNN	80.3	81.2
2.17	Medium KNN	78.7	75.0
2.18	Coarse KNN	78.7	75.0
2.19	Cosine KNN	78.7	75.0
2.20	Cubic KNN	78.7	75.0
2.21	Weighted KNN	80.3	75.0
2.22	Ensemble—boosted trees	78.7	75.0
2.23	Ensemble—bagged trees	82.0	81.2
2.24	Ensemble subspace discriminant	83.6	68.8
2.25	*Ensemble—Subspace KNN*	*85.2*	87.5
2.26	*Ensemble—RUSBoosted trees*	*85.2*	87.5
2.27	Narrow Neural Network	83.6	68.8
2.28	Medium Neural Network	83.6	75.0
2.29	*Wide Neural Network*	*85.2*	81.2
2.30	Bilayered Neural Network	83.6	75.0
2.31	**Trilayered Neural Network**	***85.2***	**93.8**
2.32	SVM kernel	80.3	81.2
2.33	Logistic Regression kernel	78.7	75.0
2.34	*Fine tree*	*85.2*	75.0
